# Determining the pathogenicity of *CFTR* missense variants: Multiple comparisons of *in silico* predictors and variant annotation databases

**DOI:** 10.1590/1678-4685-GMB-2018-0148

**Published:** 2019-11-14

**Authors:** Marcus Michels, Ursula Matte, Lucas Rosa Fraga, Aline Castello Branco Mancuso, Rodrigo Ligabue-Braun, Elias Figueroa Rodrigues Berneira, Marina Siebert, Maria Teresa Vieira Sanseverino

**Affiliations:** 1 Programa de Pós-Graduação em Genética e Biologia Molecular, Departamento de Genética, Universidade Federal do Rio Grande do Sul (UFRGS), Porto Alegre, RS, Brazil.; 2 Centro de Pesquisa Experimental, Hospital de Clínicas de Porto Alegre (HCPA), Porto Alegre, RS, Brazil.; 3 Departamento de Ciências Morfológicas, Instituto de Ciências Básicas da Saúde, Universidade Federal do Rio Grande do Sul, Porto Alegre, RS, Brazil.; 4 Grupo de Pesquisa e Pós-Graduação, Hospital de Clínicas de Porto Alegre, RS, Brazil.; 5 Programa de Pós-Graduação em Biologia Celular e Molecular, Centro de Biotecnologia, Universidade Federal do Rio Grande do Sul, Porto Alegre, RS, Brazil.; 6 Faculdade de Medicina, Universidade Federal do Rio Grande do Sul, Porto Alegre, RS, Brazil.; 7 Programa de Pós-Graduação Ciências em Gastroenterologia e Hepatologia, Faculdade de Medicina, Universidade Federal do Rio Grande do Sul, Porto Alegre, RS, Brazil.; 8 Serviço de Genética Médica, Hospital de Clínicas de Porto Alegre, Porto Alegre, RS, Brazil.; 9 Escola de Medicina, Pontifícia Universidade Católica do Rio Grande do Sul (PUCRS), Porto Alegre, RS, Brazil.

**Keywords:** CFTR, missense variant, prediction, bioinformatics, cystic fibrosis

## Abstract

Pathogenic variants in the Cystic Fibrosis Transmembrane Conductance Regulator gene (*CFTR*) are responsible for cystic fibrosis (CF), the commonest monogenic autosomal recessive disease, and *CFTR*-related disorders in infants and youth. Diagnosis of such diseases relies on clinical, functional, and molecular studies. To date, over 2,000 variants have been described on *CFTR* (~40% missense). Since few of them have confirmed pathogenicity, *in silico* analysis could help molecular diagnosis and genetic counseling. Here, the pathogenicity of 779 *CFTR* missense variants was predicted by consensus predictor PredictSNP and compared to annotations on CFTR2 and ClinVar. Sensitivity and specificity analysis was divided into modeling and validation phases using just variants annotated on CFTR2 and/or ClinVar that were not in the validation datasets of the analyzed predictors. After validation phase, MAPP and PhDSNP achieved maximum specificity but low sensitivity. Otherwise, SNAP had maximum sensitivity but null specificity. PredictSNP, PolyPhen-1, PolyPhen-2, SIFT, nsSNPAnalyzer had either low sensitivity or specificity, or both. Results showed that most predictors were not reliable when analyzing *CFTR* missense variants, ratifying the importance of clinical information when asserting the pathogenicity of *CFTR* missense variants. Our results should contribute to clarify decision making when classifying the pathogenicity of *CFTR* missense variants.

## Introduction

Cystic Fibrosis Transmembrane Conductance Regulator gene (*CFTR*; *ABCC7*; MIM #602421) (Riordan *et al.*, 1989; Rommens *et al.*, 1989) encodes for a transmembrane channel that regulates the chloride flow in the apical domain of epithelial cells. This protein is a member of the ATP-binding cassette (ABC) superfamily (Holland *et al.*, 2003). It has two membrane-spanning domains (MSD1 and MSD2), two nucleotide-binding domains (NBD1 and NBD2), and one intrinsically disordered region, the regulatory domain (RD) (Holland *et al.*, 2003; Gadsby *et al.*, 2006). The amount and/or function of the CFTR protein in the cells can be affected by disease-causing variants in the *CFTR* gene. When this channel is impaired, its malfunction damages the tissues and organs where *CFTR* expression is critical, leading to cystic fibrosis (CF; MIM #219700) – the most frequent monogenic autosomal recessive inherited disease – and *CFTR*-related disorder (Cutting, 2015).

To date, more than 2,000 variants have been described in the *CFTR* gene according to the Cystic Fibrosis Mutation Database – CFTR1 (CFTR1, 1989). Although p.Phe508del (c.1521_1523delCTT), commonly known as ΔF508, is the most common pathogenic variant in CF patients, present in about 70% of CF alleles worldwide, the ones that cause amino acid substitutions correspond to almost 40% of *CFTR* variants (Cutting, 2015; Brennan and Schrijver, 2016). Even though most missense variants are rare, several may have clinical significance. Unfortunately, the minority of them has conclusive clinical data of pathogenicity (CFTR2, 2011)[Bibr B42] [Bibr B43] [Bibr B44].

In 2015, the American College of Medical Genetics and Genomics (ACMG) and the Association for Molecular Pathology (AMP) published guidelines for the clinical laboratory interpretation of genetic variants regarding monogenic and mitochondrial diseases (Richards *et al.*, 2015). The journal cites a plethora of evidence that should be taken into consideration when establishing the pathogenicity of a genetic variant. Amid them, computational (*in silico*) predictive programs can have an auxiliary role on variant interpretation (Richards *et al.*, 2015). Among the main categories of *in silico* predictors are those that evaluate missense variants. The impact of these variants depends on criteria such as the functional consequence of the amino acid substitution, the location and the context within the protein structure and/or the evolutionary conservation of a nucleotide or amino acid. The algorithms used by those predictors consider one or more of the criteria above when assessing the impact of a missense variant (Tavtigian *et al.*, 2008; Hicks *et al.*, 2011; Thusberg *et al.*, 2011; Thompson *et al.*, 2013; Bendl *et al.*, 2014; Richards *et al.*, 2015).

Several studies have been published throughout the years that aimed at comparing the performance of *in silico* predictors and to evaluate their ability to correctly predict disease-causing variants for different genes (Tavtigian *et al.*, 2008; Dorfman *et al.*, 2010; Hicks *et al.*, 2011; Thusberg *et al.*, 2011; Thompson *et al.*, 2013; Bendl *et al.*, 2014; Bendl *et al.*, 2016). Generally speaking, the accuracy of the predictors ranges from 65 to 80% when analyzing pathogenic variants. Furthermore, most predictors tend to have low specificity, which results in an overrepresentation of these missense variants as deleterious. Also, these predictions may not be reliable when analyzing missense variants with mild effect (Thusberg *et al.*, 2011; Choi *et al.*, 2012). As an example of the clinical applicability of these predictors, different potentially deleterious SNPs in the *GBA1* gene were identified that could be associated with Gaucher’s disease (Manickam *et al.*, 2014). Specifically for CF, three common predictors (SIFT, PANTHER, and PolyPhen) were evaluated by comparing the predicted pathogenicity against the diagnosis of CF and its clinical manifestations in cohorts of subjects with CF and *CFTR*-related disorders carrying those variants (Dorfman *et al.*, 2010).

Therefore, the aim of this study was to predict the effect of *CFTR* missense variants and compare the results to public clinical data available in variant annotation databases (CFTR2 and ClinVar), verifying whether the chosen predictors would be suitable for analyzing *CFTR* missense variants, if any. In addition, we aimed to find out if there is any particular feature or modification in the CFTR protein structure that could make predictors agree or disagree more.

## Materials and Methods

### Data collection

**Figure 1:**
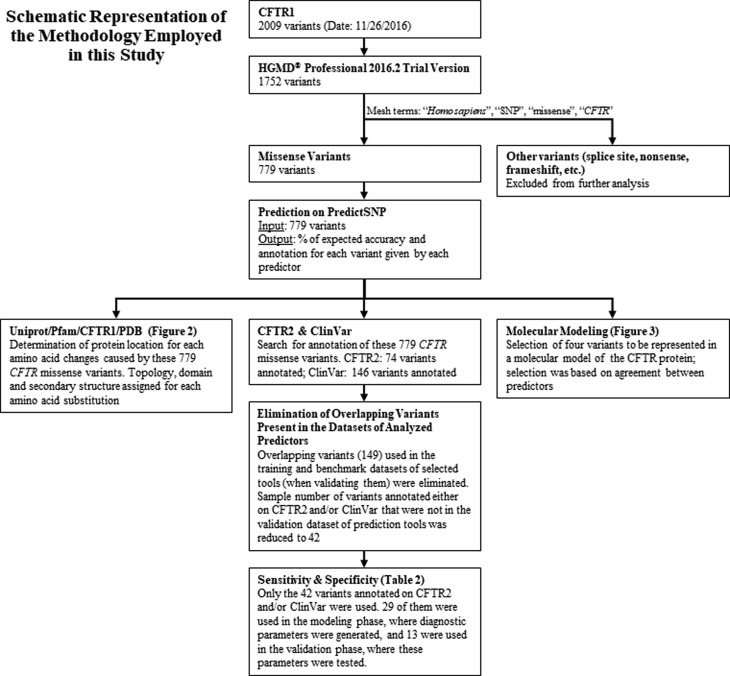
Schematic representation of the methodology employed in this study**.** Through steps of selection of variants, comparison to annotations on CFTR2 and ClinVar, and assignment of CFTR structure to each variant, two-phase sensitivity and specificity analysis and modeling of CFTR structure was performed as shown. CFTR1: Cystic Fibrosis Mutation Database; HGMD: Human Gene Mutation Database; SNP: single nucleotide polymorphism; UniProt: Universal Protein Resource; PDB: Protein Data Bank; ROC: Receiver operating characteristic curve.

A summary of this study’s workflow is represented in Figure 1. From the date this analysis started (November 2016), there were 2,009 variants described at the Cystic Fibrosis Mutation Database (CFTR1, 1989). In this study, we evaluated only variants that cause amino acid substitution; thus, 779 missense variants in the *CFTR* gene (NM_000492.3, LRG_663, ENSG00000001626) were collected from the Human Gene Mutation Database^®^ (HGMD) Professional 2016.2 Trial Version for posterior prediction of pathogenicity. This study was approved by the Hospital de Clínicas de Porto Alegre (HCPA) Ethics Committee (CAAE 59458516.5.0000.5327; GPPG 16-0644).

### Prediction

In order to predict the effect of *CFTR* missense variants in the protein, we employed the consensus classifier PredictSNP (Bendl *et al.*, 2014). The canonical protein sequence for the analysis was retrieved from the UniProt database – UniProtKB, Isoform 1 (The UniProt Consortium, 2017). PredictSNP comprises scores from different predictors (MAPP, PhDSNP, PolyPhen-1, PolyPhen-2, SIFT, SNAP, nsSNPAnalyzer, and PANTHER) and uses the information of six of them (MAPP, PhDSNP, PolyPhen-1, PolyPhen-2, SIFT, SNAP) to create its own score. PredictSNP then classifies variants as “neutral” or “deleterious” and transforms the individual confidence scores of each predictor into one comparable scale ranging from 0–100%, which represents the percentage of expected accuracy, as described elsewhere (Bendl *et al.*, 2014). By doing this, PredictSNP homogenizes the analysis. Importantly, the authors of PredictSNP constructed three independent datasets where they removed all duplicities, inconsistencies, and variants previously used in the training of the evaluated tools and a benchmark dataset containing over 43,000 variants in order to evaluate, without bias, the eight established prediction tools mentioned above. Specific methodological details are described elsewhere (Bendl *et al.*, 2014). Variants were inputted from codons 1 to 1480 using their legacy names, e.g. S1251N (c.3752G > A; p.Ser1251Asn), and then submitted to prediction analysis.

### Variant annotation databases

In order to compare the predicted pathogenicity of missense variants to the literature, we used data from CFTR2 and ClinVar as a reference to determine if predictors asserted the pathogenicity correctly.

The Clinical and Functional Translation of *CFTR* (CFTR2) is an online database for health professionals and patients, gathering clinical, molecular, and functional information of CF (CFTR2, 2011). Also, it publishes at least once a year a list of curated variants already found in patients across the globe. CFTR2 classifies variants as “CF-causing”, “Non CF-causing”, “Varying clinical consequence”, and “Unknown significance”. Sometimes, a variant may change from one class to another in an updated version of this list. For this study, we used the most up-to-date list available on CFTR2 (CFTR2_17March2017.xlsx) when we were gathering data as a reference to compare the predicted and the clinical information of pathogenicity. Only the minority of variants that we analyzed on PredictSNP were recorded in the CFTR2 list (74 variants; 9.5%).

The other variant annotation database, ClinVar, is a platform of the National Center for Biotechnology Information (NCBI) that aggregates information about genomic variation and its relationship to human health (Landrum *et al.*, 2018). Regarding Mendelian diseases, ClinVar uses the five standard terms to classify the clinical significance of variants according to Richards *et al.* (2015), classifying them as “Pathogenic”, “Likely pathogenic”, “Unknown significance”, “Likely benign”, and “Benign”. Different sources can submit data of any variant of the human genome, but not all data on ClinVar is curated. Only 146 of the 779 (18.7%) *CFTR* missense variants analyzed in this study were described on ClinVar until November 20, 2016, which is the date when we did the research in the website. Also, CFTR2 submits variant data regarding the *CFTR* gene to ClinVar.

### CFTR topology, domains, and secondary structure

Information about CFTR structure was gathered from different sources. Data from the CFTR protein topology were retrieved from the UniProt database – UniProtKB (The UniProt Consortium, 2017), and divided into “cytoplasmic”, “transmembrane”, and “extracellular”, according to the amino acid position in relation to the cell membrane. The information about CFTR domains (MSD1, NBD1, RD, MSD2 and NBD2) was collected both from Pfam (Finn *et al.*, 2016) and CFTR1. When data diverged between them, CFTR1 data were chosen since it is a specific database for the *CFTR* gene. Regarding the secondary structure of CFTR, information was collected from the Protein Data Bank (RCSB PDB) [Bibr B45] [Bibr B46] (Berman *et al.*, 2000), using the PDB ID: 5UAK (Liu *et al.*, 2017). Features represented in the secondary structure of CFTR were divided according to RCSB PDB into: “β-strand”, “turn”, “empty (no secondary structure assigned)”, “3/10-helix”, “β-bridge”, “bend”, and “α-helix”.

### Modeling of CFTR protein and possible effect of elected variants

The structural modeling of the CFTR protein (UniProtKB number: P13569) was performed using the I-TASSER package (Zhang, 2008; Roy *et al.*, 2010; Yang *et al.*, 2015). Through sequential steps of identification of possible template structures, template fragmentation, incremental model construction and evaluation, the tool was able to construct a high-quality model for the protein (residues 1-1480). The visualization of the structures was performed with the software PyMOL (The PyMOL Molecular Graphics System, Version 1.8 Schrödinger, LLC). This model was created to verify the possible implications of four different *CFTR* missense variants that were chosen based on the agreement or disagreement shown by all predictors for each one of them. The variants picked for the model were: p.Met1Val (c.1A > G), p.Arg117His (c.350G > A), p.Gly551Asp (c.1652G > A), and p.Ile1027Thr (c.3080T > C).

### Sensitivity and specificity analysis

In order to compare predictions to annotations available on CFTR2 and/or ClinVar, annotated variants that were used to validate (present in the training or benchmark dataset) of any predictor were excluded from the analysis (ure 1). Therefore, from the variants annotated on CFTR2 and/or ClinVar, only 42 remained for further evaluation, allowing for an unbiased comparison of predictor performance. These remaining 42 variants were randomly divided in two phases, each one composed by deleterious and neutral variants: 1) Modeling phase: 29 out of 42 variants were randomly used to build a ROC curve, where sensitivity and specificity were calculated; 2) Validation phase: the remaining 13 variants (nine CF-causing and four Non-CF-causing) were used to verify if parameters generated in the modeling phase were trustworthy (Table S1). For this analysis, accuracy values were used as a continuous variable. Variants predicted as neutral were analyzed as negative accuracies, differentiating them from those accuracies of variants predicted as deleterious. Hence, a continuous variable ranging from -1 to +1 (absolute frequency of the percentage of expected accuracy) was used.

### Statistical analysis

For this study, “Non CF-causing”, “Benign”, and “Likely benign” variants were considered “neutral” while “CF-causing”, “Pathogenic” and “Likely pathogenic” variants were considered “deleterious”. For the sensitivity and specificity analysis, Youden Index J was employed in the modeling phase to determine the cut-off threshold where sensitivity and specificity parameters would be maximized, generating the best possible diagnostic parameters (Youden, 1950). In order to verify if there was any amino acid change, any particular region, domain, or any secondary feature of the CFTR protein that was associated with a higher or lower agreement between predictors, Pearson’s Chi-Squared or Fisher’s Exact Test was used as appropriate. We counted the agreement or disagreement between predictors based on the predicted pathogenicity (“neutral” or “deleterious”) for a given variant, as follows: “0” (full agreement), “1” (1 disagreement), “2” (2 disagreements), and “3” (3 disagreements). Predictors that could not assign a prediction to any of the 779 *CFTR* missense variants (then considering them as “missing”) were excluded from the analysis (namely, MAPP and PANTHER) (e 1). Results were analyzed using SPSS v18.0. Data were considered statistically significant when *p* < 0.05.

## Results

### Descriptive analysis

Descriptive data of variants submitted to prediction analysis are shown in Table 1. All *in silico* tools predicted the pathogenicity of the 779 *CFTR* missense variants except for MAPP (missing = 53) and PANTHER (missing=488). At least 25% of the predictions made by PredictSNP had an accuracy of 87%. Also, nsSNPAnalyzer had the lowest amplitude and its predictions had lower accuracy than other predictors. At least half of the accuracies provided by nsSNPAnalyzer were of 63%.

**Table 1:** Descriptive analysis of each predictor for *CFTR* missense variants (n=779^a^).

Predictor		PredictSNP^e^	MAPP	PhDSNP	PolyPhen1	PolyPhen2	SIFT	SNAP	nsSNPAnalyzer	PANTHER
Number of predicted variants used in location analysis	Valid	779	726	779	779	779	779	779	779	291
	Missing	0	53	0	0	0	0	0	0	488
Number of variants used in sensitivity and specificity analysis	Valid	42	39	42	42	42	42	42	42	-
	Missing	0	3^b^	0	0	0	0	0	0	-
Pathogenicity	Neutral (%)^c^	286 (36.7%)	339 (46.7%)	218 (28.0%)	400 (51.3%)	287 (36.8%)	211 (27.1%)	264 (33.9%)	292 (37.5%)	192 (66.0%)
Deleterious (%)		493 (63.3%)	387 (53.3%)	561 (72.0%)	379 (48.7%)	492 (63.2%)	568 (72.9%)	515 (66.1%)	487 (62.5%)	99 (34.0%)
Mean of Expected Accuracy^d^ (SD)		73 (11.8)	69 (12.3)	73 (12.6)	67 (5.2)	66 (13.9)	71 (13.0)	70 (12.0)	64 (1.0)	61 (7.9)
Minimum		51	41	45	59	40	43	50	63	47
Maximum		87	92	98	74	87	90	89	65	74
Percentiles	25	63	62	61	67	55	65	61	63	56
	50 (Median)	74	72	77	67	68	79	72	63	63
	75	87	77	86	74	81	79	81	65	68

a Missense variants were retrieved from HGMD^®^ Professional 2016.2 Trial Version on 09/29/2016.

b MAPP could not assign a prediction for three out of the 42 variants used for the sensitivity and specificity analysis (Supp. Table S1).

c Parenthesis represent the percentage of neutral or deleterious predictions over the valid number of predicted variants.

d Expected accuracy is a comparable scale ranging from 0–100% which represents the transformed confidence scores of individual predictors.

e PredictSNP uses scores from MAPP, PhDSNP, PolyPhen1, PolyPhen2, SIFT and SNAP to create its own prediction scores.

Descriptive data of each predictor are depicted. Missing values represent that predictors were not able to assign the pathogenicity of a variant. SD: Standard deviation.

### Sensitivity and specificity analysis

In the modeling phase of the sensitivity and specificity analysis, ROC curves and the best possible cut-off thresholds were generated, as evidenced in Table 2. In the validation phase, the remaining variants generated diagnostic parameters that could better indicate the performance of each predictor. Noteworthy, MAPP’s modeling phase was constituted by 22 deleterious and 4 neutral variants, since the other 3 variants belonged to the group of 56 missing variants that MAPP could not attribute a prediction result, namely, p.Asp836Tyr (CF-causing), p.Asn900Lys (CF-causing), and p.Arg668Cys (Non CF-causing).

**Table 2:** Best cut-off threshold of each predictor in the sensitivity and specificity analyses (n=42).

Best cut-off threshold of each predictor according to variant annotation databases in the modeling phase (n=29)						Sensitivity and specificity of each predictor according to variant annotation databases in the validation phase (n=13)	
Predictor	AUC^a^	Youden Index J	Cut-off (% of expected accuracy)	Sensitivity (%)	Specificity (%)	Sensitivity (%)	Specificity (%)
MAPP^b^	0.773	0.500	≥ 50	50	100	33.3	100
SNAP	0.658	0.400	≥ -74	100	40	100	0
PolyPhen2	0.638	0.350	≥ -66	75	60	55.6	50
PhDSNP	0.604	0.333	≥ 84	33.3	100	22.2	100
SIFT	0.575	0.317	≥ -78.5	91.7	40	77.8	0
PolyPhen1	0.567	0.267	≥ -4	66.7	60	44.4	25
nsSNPAnalyzer	0.475	-0.050	≥ -1	75	20	66.7	50
PredictSNP^c^	0.613	0.358	≥ -74.5	95.8	40	77.8	25

a AUC = area under the curve representing the discrimination between variants that are and are not deleterious. It ranges from 0 to 1 (values close to 1 represent predictors with better performances).

b In the modeling phase, MAPP’s ROC curve was built using 22 deleterious and four neutral variants (n=26). The other three variants were considered “missing” in our dataset because MAPP could not generate a valid prediction for these variants (Supp. Table S1).

c PredictSNP uses scores from MAPP, PhDSNP, PolyPhen1, PolyPhen2, SIFT and SNAP to create its own prediction scores.

Negative cut-off values represent cut-offs of a neutral prediction.

Youden Index J (Youden, 1950) is a statistical test to rate diagnostic tests, ranging from 0 to 1 (values close to 1 represent predictors with better performances). Youden Index is calculated according to the equation: J = (Sensitivity + Specificity) - 1

PANTHER was not included in this analysis.

All data presented in this table is statistically significant (*p* < 0.05).

Overall, predictors had poor performances in this analysis. However, the two-phase experiment revealed that, at the best cut-off threshold that Youden J Index could find, MAPP and PhDSNP had maximum specificity, even though both had low sensitivities (Table 2). Interestingly, when comparing the results shown in Table 2 to our bank of missense variants, the ones annotated as CF-causing or at least having varying clinical consequence were predicted as deleterious when the expected accuracy surpassed the threshold. MAPP had two exceptions, with variants being Non CF-causing but predicted as deleterious. Although SNAP had maximum sensitivity in the validation phase (Table 2), it presented null specificity. The same specificity result applies to SIFT. This result was not relevant since all 13 variants in the validation phase were predicted as deleterious.

### Agreement between predictors according to CFTR structure

**Figure 2:**
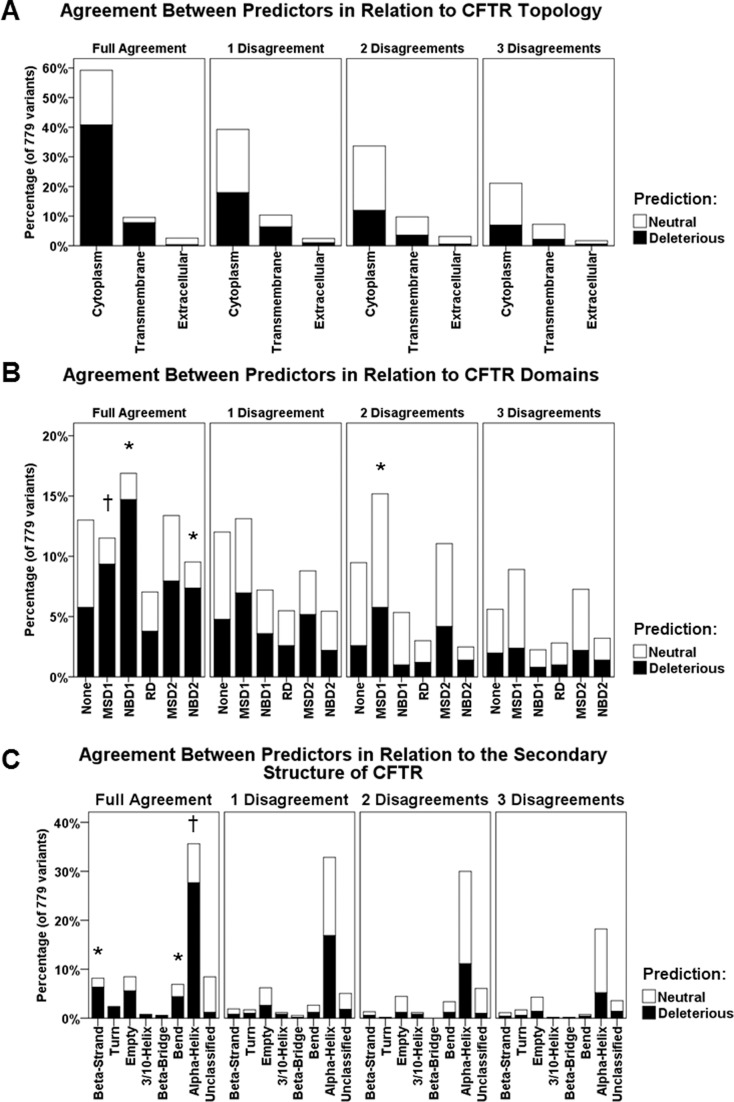
Agreement between predictors according to CFTR protein structure. A) Agreement between predictors in relation to CFTR topology. Even though there was a tendency of association between full agreement and cytoplasm, it was not statistically significant (*p*=0.052). B) Agreement between predictors in relation to CFTR domains. NBD1 and NBD2 were significantly associated with full agreement between predictors. MSD1 was not significantly associated with full agreement and significantly associated with two disagreements between predictors (*p* < 0.001). C) Agreement between predictors in relation to the secondary structure of CFTR. Beta-strands and bends were significantly associated with full agreement between predictors whilst alpha-helices were not. Overall, predictors tended to agree more when they predicted missense variants as deleterious (black columns). As the disagreements increased, the neutral prediction got more frequent (white columns). *p < 0.05; : *p* > 0.05. MAPP and PANTHER were not included in this analysis.

We wanted to evaluate the agreement between predictors in relation to CFTR’s topology (Figure 2A), domains (Figure 2B), and features of secondary structure (Figure 2C). MAPP and PANTHER were excluded from this analysis because they were not capable of assigning a prediction for a considerable number of *CFTR* missense variants. The results showed that predictors have the tendency to fully agree when amino acid changes are located in the cytoplasm, although not statistically significant (Figure 2A; *p*=0.052). To what concerns protein domains, CFTR amino acid changes located in NBD1 and NBD2 were significantly associated with full agreement between predictors (*p* < 0.001), as expected. For those changes located in MSD1, on the contrary, predictors tended to not agree fully, being directly associated with two disagreements between predictors (Figure 2B; *p* < 0.001). Taking the features of CFTR secondary structure into account, amino acid substitutions located in β-strands and bends were associated with full agreement between predictors whereas amino acid changes in α-helices were associated with at least one disagreement between predictors (Figure 2C; *p*=0.001).

### Variants in the CFTR protein model

**Figure 3:**
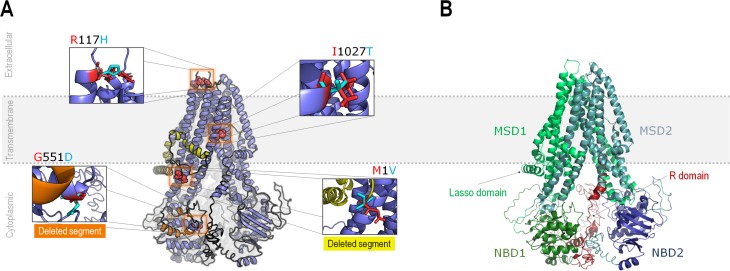
Modeled structure of the CFTR protein. A) Modeled structure for CFTR in its proposed location when inserted in biomembranes (dotted lines), evidencing the absence of extracellular domains. Proposed variants are highlighted, while two segments that may be lost (at least partially) as a function of these variants are highlighted in orange (associated with p.Met1Val) and in yellow (associated with p.Arg117His). Details regarding these deletions are given in the results session. B) Main structural features of human CFTR. The semi-symmetric structure is composed of two equivalent halves, comprising two domains (MSD and NBD) each. The N-terminus half of the protein has exclusive topological regions, namely the Lasso domain and the R domain (or R insertion). MSD: Membrane-spanning domain; NBD: Nucleotide-binding domain; R domain: Regulatory domain.

Examples of variants that would be featured in the CFTR protein model (Figure 3) were chosen based on the agreement between predictors, as described in the next paragraphs.

Predictors fully agreed that p.Met1Val (c.1A > G; legacy name M1V) is a neutral variant. However, our model (Figure 3A) showed that this variant would deviate the translation initiation to the second methionine codon in the mRNA molecule, causing the loss of the first 81 amino acids of the protein, which is corroborated by data available on CFTR2 and ClinVar.

One example of a variant that showed disagreement between predictors is p.Arg117His (c.350G > A; legacy name R117H). Considering that the residue of arginine (positively charged at physiological pH) is preceded by two glutamic acids (negatively charged at physiological pH), its substitution for histidine, which has an imidazole ring in the side chain and is also positively charged at physiological pH, could disturb the local neutralization. Besides, codon 117 is in the interface between transmembrane and extracellular segments of the protein, which could also cause a local disturbance (Figure 3A). Since p.Arg117His is well known for being pathogenic when in *cis* with the 5T (c.1210-12[5]) allele of the poly-T tract (c.1210-12[5-9]), which causes the skipping of exon 10 during mRNA processing, we also elaborated a model contemplating the effect of both *CFTR* variants when in *cis* (not shown). The combination p.[Arg117His;5T] (c.[350G > A;1210-12[5]]) in the same CFTR model did not offer conclusive results of its pathogenicity, which corroborates the disagreement verified in the analysis of *in silico* predictors.

Another chosen variant was p.Gly551Asp (c.1652G > A; legacy name G551D), which had 100% agreement between predictors as being deleterious. In our model (Figure 3A), the p.Gly551Asp could change the local molecular environment of the NBD1 domain, establishing new interactions, by replacing an amino acid that lacks a side chain with a negatively charged one (at physiological pH), which may be the cause of its pathogenicity.

The last variant picked from our database was p.Ile1027Thr (c.3080T > C; legacy name I1027T). This variant changes isoleucine (hydrophobic) for threonine (polar and uncharged at physiological pH) in the middle of a transmembrane helix, where the side chain is exposed to the lipid portion of the cell membrane. This variant was considered neutral by all predictors with the exception of Polyphen1, and also considered benign by CFTR2 and ClinVar.

## Discussion

In this study, we submitted 779 *CFTR* missense variants to prediction analysis in the consensus classifier PredictSNP. We compared prediction results to annotations available on CFTR2 and ClinVar in order to determine if any of these predictors would present a reliable interpretation of these variants.

Analysis of sensitivity and specificity showed that none of the predictors had a reliable performance predicting the pathogenicity of *CFTR* missense variants. It was observed that MAPP and PhDSNP had maximum specificity, correctly identifying true negatives, i.e., non-pathogenic variants. However, the fact that the sensitivity of both predictors was lower than 50% means that they would be randomly assigning variants as deleterious. It is important to highlight that a higher specificity would be preferred instead of a higher sensitivity, since molecular diagnosis is not used for screening but as a complementary form of CF diagnosis, where the clinical features and the sweat chloride test have already been performed in patients (Farrell *et al.*, 2017). Hence, the goal would be to avoid predicting neutral variants as pathogenic. On the other hand, both SIFT and SNAP presented good sensitivity in the detriment of a null specificity. This result is a clear example of one of the main struggles of *in silico* predictors: overprediction of variants as deleterious (Choi *et al.*, 2012; Richards *et al.*, 2015).

One factor that may contribute to the disagreements between the prediction of predictors is the algorithms employed by each one of these tools (Bendl *et al.*, 2014). Four predictors included in the analysis generated by PredictSNP – PhDSNP (Capriotti *et al.*, 2006), PolyPhen2 (Adzhubei *et al.*, 2010), SNAP (Bromberg and Rost, 2007), nsSNPAnalyzer (Chandonia *et al.*, 2004; Bao *et al.*, 2005) – apply machine-learning methods to train their decision models (Larrañaga *et al.*, 2006). From the other predictors, SIFT (Ng and Henikoff, 2003) and PANTHER (Thomas *et al.*, 2003; Brunham *et al.*, 2005) use only evolutionary information while MAPP (Stone and Sidow, 2005) also considers differences in the physicochemical properties of wild type and mutated amino acids in their prediction. PolyPhen1 (Ramensky *et al.*, 2002) uses a set of empirical rules in order to classify missense variants. This diversity in the way predictors analyze missense variants indicates that these tools should not be used as the only way to assert the pathogenicity of this type of variant. Besides, multiple lines of computational evidence that support a deleterious or a benign impact on a gene or gene product should not be counted as an independent criterion, therefore being counted only once in any evaluation of a variant (Richards *et al.*, 2015).

Considering the structure of the CFTR protein, we had some interesting findings. Regarding the agreement between predictors in relation to neutral or deleterious predictions, we observed that missense variants located in nucleotide-binding domains (NBD1 and NBD2), β-strands, and bends are associated with full agreement between computational tools (Figure 2). One explanation for this resides in the fact that all β-strands present in the CFTR structure are located either in NBD1 or NBD2. Notably, the CFTR protein is a peculiar member of the ABC superfamily (Holland *et al.*, 2003; Gadsby *et al.*, 2006) and it is fundamentally composed by two halves, each half having one membrane-spanning domain (MSD) and one NBD (Figure 3B). Otherwise, missense variants located in MSD1 and in α-helices are associated with at least one disagreement between predictors (Figure 2). Herein, not just MSD1 and MSD2 are basically formed by α-helices but this secondary structure is also present in other domains. The structure 5UAK curated in the Protein Data Bank (Berman *et al.*, 2000; Liu *et al.*, 2017), used as a reference to determine where the amino acid substitution generated by each *CFTR* missense variant is located in the secondary structure, corroborates the data above. At least visually, our results suggest that predictors tend to agree more when they assert variants as deleterious, and they also tend to disagree more when asserting variants as neutral.

The molecular model elaborated with p.Met1Val, p.Arg117His, p.Gly551Asp, and p.Ile1027Thr made it possible to better rationalize the effect of these variants in the CFTR protein, and some of the affirmations generated by our model were corroborated by CFTR2 and ClinVar data. In the case of p.Met1Val, translation initiation at the first methionine would be aborted, promoting the loss of the first 81 amino acids of the protein sequence (Figure 3B), which includes the Lasso domain (Liu *et al.*, 2017). Our analysis is corroborated by CFTR2 and ClinVar, where p.Met1Val is classified, respectively, as “CF-causing” and “Pathogenic”. In fact, there were 26 patients in the CFTR2 database that carried this pathogenic variant (CFTR2, 2011).

A variant that showed disagreement between predictors is p.Arg117His (c.350G > A; legacy name R117H), which is described as having varying clinical consequence by CFTR2. In the same database, there are 1,817 patients that carry p.Arg117His (CFTR2, 2011). According to ClinVar, this variant is pathogenic, has conflicting interpretation of pathogenicity, and is also a risk factor. In addition, when p.Arg117His is in *cis* with c.1210-12[5] (5T form of the poly-T tract, an intragenic modifier that causes the skipping of exon 10 during mRNA processing), this combination is considered as CF-causing according to CFTR2, being carried by 102 patients (CFTR2, 2011). It is important to emphasize that p.Arg117His and c.1210-12[5] do not cause CF when they are alone or in *trans* (CFTR2, 2011). According to our model, the combination c.[350G > A;1210-12[5]] does not offer conclusive results of its pathogenicity, which corroborates curated data (CFTR2, 2011), but reinforces the inconclusive predictions of *in silico* tools. Finally, the amino acid change generated by this missense variant affects the function of the CFTR protein. When arginine is substituted by a histidine, the conductance of CFTR is affected, thus impairing the flow of chloride ions (Sheppard *et al.*, 1993). Ivacaftor (Kalydeco^®^; Vertex Pharmaceuticals Inc., Boston, MA), a drug approved to treat gating defects caused by *CFTR* missense variants, has already been approved by the U.S. Food and Drug Administration for the treatment of patients carrying p.Arg117His as well (Vertex Pharmaceuticals Inc.).

The substitution of a glycine for an aspartate on codon 551 (p.Gly551Asp; c.1652G > A; legacy name G551D) has been reported as CF-causing by CFTR2 and pathogenic by ClinVar. This variant does not affect the amount of CFTR protein available in the cell membrane. Instead, its pathogenicity relies upon the functional activity of CFTR, impairing the gating of this chloride channel due to its proximity with the ATP-binding site. The p.Gly551Asp variant was also chosen to be featured in our model (Figure 3A) because it is one of the two *CFTR* missense variants carried by more than 1% of CF patients (Cutting, 2015; Brennan and Schrijver, 2016), being a well-known therapeutic target of ivacaftor. In fact, there are 2,915 patients carrying this variant in the CFTR2 database.

The last variant picked from our database was p.Ile1027Thr (c.3080T > C; legacy name I1027T). Although this substitution could affect the permeability of the CFTR channel, it was considered neutral by all predictors except for PolyPhen1. Concerning CFTR2, p.Ile1027Thr is a non-CF causing variant. This annotation was based on clinical information of patients carrying this variant, experimental data generated from this variant, and on groups of healthy individuals that carry p.Ile1027Thr. CFTR2 also reports that there are 36 patients carrying this variant in its database. Concerning ClinVar, p.Ile1027Thr is a benign or likely benign variant.

One important limitation that we encountered was the lack of missense variants reported in the variant annotation databases. When the comparison between predictions and variant annotation databases started, the most updated list of *CFTR* variants available on CFTR2 (“CFTR2_17March2017.xlsx”), a curated database specific to the *CFTR* locus, contained the 322 most common variants, and approximately 80 were missense. Currently, the up-to-date list of *CFTR* variants (CFTR2_8December2017_2.xlsx) contains the 374 most common variants in the *CFTR* gene, and the number of missense variants has increased to almost 120. It represents an improvement concerning the intrinsic difficulty of making functional analyses to evaluate the activity of this chloride channel and the rarity of most *CFTR* missense variants in the population. Besides, CFTR2 only presents information about variants that have been reported in one of the 88,664 patients currently registered in the database (CFTR2, 2011). Regarding ClinVar, variant classification is provided by different submitters (CFTR2 being one of them). In this database, about 600 *CFTR* missense variants have available information about their pathogenicity, which would increase the sample size of annotated variants in this study. However, almost half of them are variants of unknown significance (VUS), and there are cases of conflicting interpretation of pathogenicity, many of them among the 779 missense variants submitted to prediction analysis in this study. This limitation had an impact on the sample size of our validation, since most reported variants had already been used in the training datasets of the evaluated predictors.

Since we chose predictors that were available on PredictSNP as a model, the inclusion of other predictors would require a sophisticated mathematical study, which would deviate from the clinical/practical scope of this study. Although there are more current predictors available, like CADD, MutPred, VEST, FATHMM, REVEL (Ioannidis *et al.*, 2016), the ones that were included in our study are in fact listed by Richards and colleagues as suitable predictors to evaluate the pathogenicity of missense variants in monogenic and mitochondrial diseases (Richards *et al.*, 2015).

These results ratified that predictors not only diverge when predicting the pathogenicity of these variants but can also agree to assign the wrong annotation to variants that clearly have the opposite effect, mainly corroborating previous studies that showed the low specificity of *in silico* predictors and the overprediction of deleterious variants (Choi *et al.*, 2012; Richards *et al.*, 2015). Overall, the categorical classification as “neutral” or “deleterious” for missense variants in a gene that encodes for a transporter, which function can range from zero to 100%, poses significant limitation for its use. Hence, i*n silico* analysis, as part of the molecular analysis of *CFTR*, should always be correlated with clinical – signs and symptoms – and physiological data in order to determine CF diagnosis. Concomitantly, the further determination of pathogenicity and the reevaluation of missense variants curated in annotation databases like CFTR2 are fundamental, mainly because there are those cases of positive newborn screening, inconclusive diagnosis, and CFTR-related metabolic syndrome (Farrell *et al.*, 2017), where CF diagnosis is very difficult to achieve.

This study employed a consensus predictor to evaluate a large number of *CFTR* missense variants and compare these predictions to publicly available variant annotation databases (CFTR2 and ClinVar). As shown by the results presented in previous sections, predictors should be used carefully under a critical point of view, since *in silico* data are only a supporting evidence of pathogenicity. They have less power as an evidence when classifying a variant than clinical and population data (Richards *et al.*, 2015). Further studies and validation of other predictors are necessary in order to identify prediction tools more suitable for helping clinicians and genetic counselors on decision making about the pathogenicity of *CFTR* missense variants.

## Supplementary material

The following online material is available for this article:

Table S1 Annotated variants included in the sensitivity and specificity analysis.
