# Supplementary material for: Determining the pathogenicity of CFTR missense variants: Multiple comparisons of in silico predictors and variant annotation databases
Source: Genet Mol Biol. 2019 Nov 14;42(3):560–70. doi: 10.1590/1678-4685-GMB-2018-0148 (PMC6905453; doi:10.1590/1678-4685-GMB-2018-0148)
Supplement: Supplementary file 1 [file 1415-4757-GMB-42-3-2018-0148-suppl1.pdf]

Supplementary Material to "Determining the pathogenicity of CFTR missense variants: Multiple comparisons of in silico predictors and variant annotation databases"

Table S1 - Annotated variants included in the sensitivity and specificity analysis.

**Modeling phase (n=29)**		**Validation phase (n=13)**	
**Deleterious variants (n=24)**	**Neutral variants (n=5)**	**Deleterious variants (n=9)**	**Neutral variants (n=4)**
p.Met1Thr	p.Arg31Cys	p.Met1Val	p.Ile148Thr
p.Pro5Leu	p.Ile285Phe	p.Glu56Lys	p.Met348Lys
p.Ala46Asp	p.Arg668Cys[a]	p.Glu217Gly	p.Phe508Cys
p.Ile119Val	p.Ser1235Arg	p.Leu227Arg	p.Ile1027Thr
p.Arg297Trp	p.Phe1257Leu	p.Ala349Val	
p.Ser341Pro		p.Ala534Glu	
p.Thr360Lys		p.Gly622Asp	
p.Asp373Asn		p.Try1032Cys	
p.Val456Ala		p.Gln1352His	
p.Leu467Pro			
p.Ile506Met			
p.Ala561Glu			
p.Asp836Tyr[a]			
p.Asn900Lys[a]			
p.Ser912Leu			
p.Leu927Pro			
p.Val938Gly			
p.Ser945Leu			
p.Gly970yAsp			
p.Gly970Arg			
p.Pro1013His			
p.His1054Asp			
p.Phe1074Leu			
p.Gly1244Val			

[a] This variant was not predicted by MAPP. Therefore, MAPP's diagnostic parameters and ROC curve did not include this variant.
